# On Some Growth Properties of Entire Functions Using Their Maximum Moduli Focusing (*p*, *q*)th Relative Order

**DOI:** 10.1155/2014/164130

**Published:** 2014-11-03

**Authors:** Luis Manuel Sanchez Ruiz, Sanjib Kumar Datta, Tanmay Biswas, Golok Kumar Mondal

**Affiliations:** ^1^ETSID, Departamento de Matemática Aplicada & CITG, Universitat Politècnica de València, 46022 Valencia, Spain; ^2^Department of Mathematics, University of Kalyani, Kalyani, Nadia, West Bengal 741235, India; ^3^Rajbari, Rabindrapalli, R. N. Tagore Road, P.O.-Krishnagar, Nadia, West Bengal 741101, India; ^4^Dhulauri Rabindra Vidyaniketan (H.S.), Vill +P.O.-Dhulauri, P.S.-Domkal, Murshidabad, West Bengal 742308, India

## Abstract

We discuss some growth rates of composite entire functions on the basis of the definition of relative (*p*, *q*)th order (relative (*p*, *q*)th lower order) with respect to another entire function which improve some earlier results of Roy (2010) where *p* and *q* are any two positive integers.

## 1. Introduction, Definitions, and Notations

Let *f* be an entire function defined in the open complex plane and let

(1) $$\begin{array}{c} M_{f}\left(r\right)=\max\left\{\left|f\left(z\right)\right|:\left|z\right|=r\right\} \end{array}$$

be its maximum modulus function. If *f* is nonconstant then *M*_*f*_(*r*) is strictly increasing and continuous and its inverse *M*_*f*_^−1^(*r*) : (|*f*(0)|, *∞*) → (0, *∞*) exists and is such that

(2) $$\begin{array}{c} \underset{s\to \infty }{\lim}M_{f}^{-1}\left(s\right)=\infty . \end{array}$$

We use the standard notations and definitions in the theory of entire functions which are available in [1]. In the sequel we use the following notation:

(3) log⁡[0]x=x,  log⁡[k]x=log⁡log⁡[k−1]xfor  k=1,2,3,…,exp⁡[0]x=x,  exp⁡[k]x=exp⁡exp⁡[k−1]xfor  k=1,2,3,….

The following definitions are well known.

**Definition 1 .**

The order *ρ*_*f*_ and the lower order *λ*_*f*_ of an entire function *f* are defined as

(4) $$\begin{array}{c} \rho_{f}=\lim\underset{r\to \infty }{\sup}\frac{\log^{\left[2\right]}M_{f}\left(r\right)}{\log r},\\ \lambda_{f}=\lim\underset{r\to \infty }{\inf}\frac{\log^{\left[2\right]}M_{f}\left(r\right)}{\log r}. \end{array}$$

Juneja et al. [2] defined the (*p*, *q*)th order and (*p*, *q*)th lower order of an entire function *f*, respectively, as follows:

(5) $$\begin{array}{c} \rho_{f}\left(p,q\right)=\lim\underset{r\to \infty }{\sup}\frac{\log^{\left[p\right]}M_{f}\left(r\right)}{\log^{\left[q\right]}r},\\ \lambda_{f}\left(p,q\right)=\lim\underset{r\to \infty }{\inf}\frac{\log^{\left[p\right]}M_{f}\left(r\right)}{\log^{\left[q\right]}r}, \end{array}$$

where *p*, *q* are any two positive integers with *p* ≥ *q*.

If *p* = *l* and *q* = 1 then we write *ρ*_*f*_(*l*, 1) = *ρ*_*f*_^[*l*]^ and *λ*_*f*_(*l*, 1) = *λ*_*f*_^[*l*]^.

Also for *p* = 2 and *q* = 1 we, respectively, denote *ρ*_*f*_(2,1) and *λ*_*f*_(2,1) by *ρ*_*f*_ and *λ*_*f*_.

In this connection we just recall the following definition.

**Definition 2 (see [2]).**

An entire function *f* is said to have index-pair (*p*, *q*), *p* ≥ *q* ≥ 1, if *b* < *ρ*_*f*_(*p*, *q*) < *∞* and *ρ*_*f*_(*p* − 1, *q* − 1) is not a nonzero finite number, where *b* = 1 if *p* = *q* and *b* = 0 if *p* > *q*. Moreover if 0 < *ρ*_*f*_(*p*, *q*) < *∞*, then

(6) $$\begin{array}{c} \rho_{f}\left(p-n,q\right)=\infty \;\text{for}\;\;n<p,\\ \rho_{f}\left(p,q-n\right)=0\;\text{for}\;\;n<q,\\ \rho_{f}\left(p+n,q+n\right)=1\;\text{for}\;\;n=1,2,\ldots . \end{array}$$

Similarly for 0 < *λ*_*f*_(*p*, *q*) < *∞*, one can easily verify that

(7) $$\begin{array}{c} \lambda_{f}\left(p-n,q\right)=\infty \;\text{for}\;\;n<p,\\ \lambda_{f}\left(p,q-n\right)=0\;\text{for}\;\;n<q,\\ \lambda_{f}\left(p+n,q+n\right)=1\;\text{for}\;\;n=1,2,\ldots . \end{array}$$

An entire function for which (*p*, *q*)th order and (*p*, *q*)th lower order are the same is said to be of regular (*p*, *q*)-growth. Functions which are not of regular (*p*, *q*)-growth are said to be of irregular (*p*, *q*)-growth.

Bernal [3] introduced the definition of relative order of *f* with respect to *g*, denoted by *ρ*_*g*_(*f*) as follows:

(8) ρgf =inf⁡μ>0:Mfr<Mgrμ  ∀r>r0μ>0 =lim⁡sup⁡r→∞log⁡Mg−1Mfrlog⁡r.

The definition coincides with the classical one [4] if *g* = exp⁡.

Similarly one can define the relative lower order of *f* with respect to *g* denoted by *λ*_*g*_(*f*) as follows:

(9) $$\begin{array}{c} \lambda_{g}\left(f\right)=\lim\underset{r\to \infty }{\inf}\frac{\log M_{g}^{-1}M_{f}\left(r\right)}{\log r}. \end{array}$$

In the case of relative order, it therefore seems reasonable to define suitably the relative (*p*, *q*)th order of entire functions. Lahiri and Banerjee [5] also introduced such definition in the following manner.

**Definition 3 (see [5]).**

Let *p* and *q* be any two positive integers with *p* > *q*. The relative (*p*, *q*)th order of *f* with respect to *g* is defined by

(10) ρgp,qf =inf⁡μ>0:Mfr<Mgexp⁡p−1μlog⁡qr    ∀r>r0μ>0 =lim⁡sup⁡r→∞log⁡p−1Mg−1Mfrlog⁡qr.

If *q* = 1, *k* ≥ 1, and *p* = *k* + 1 then *ρ*_*g*_^(*p*, *q*)^(*f*) = *ρ*_*g*_^*k*^(*f*). If *g* = exp⁡*z* then *ρ*_*g*_^(*p*, *q*)^(*f*) = *ρ*_*f*_(*p*, *q*).

Sánchez Ruiz et al. [6] gave a more natural definition of relative (*p*, *q*)th order of an entire function in light of index-pair which is as follows.

**Definition 4 .**

Let *f* and *g* be any two entire functions with index-pairs (*m*_1_, *q*) and (*m*_2_, *p*), respectively, where *m*_1_ = *m*_2_ = *m* and *p*, *q*, and *m* are all positive integers such that *m* ≥ *p* and *m* ≥ *q*. Then the relative (*p*, *q*)th order of *f* with respect to *g* is defined as

(11) ρgp,qf =inf⁡μ>0:Mfr<Mg    ×exp⁡plog⁡m2exp⁡m1μlog⁡qr    ∀r>r0μ>0 =inf⁡μ>0:Mfr<Mgexp⁡pμlog⁡qr    ∀r>r0μ>0 =lim⁡sup⁡r→∞log⁡pMg−1Mfrlog⁡qr.

Similarly one can define the relative (*p*, *q*)th lower order of an entire function *f* with respect to another entire function *g* denoted by *λ*_*g*_^(*p*, *q*)^(*f*) where *p* and *q* are any two positive integers in the following way:

(12) $$\begin{array}{c} \lambda_{g}^{\left(p,q\right)}\left(f\right)=\lim\underset{r\to \infty }{\inf}\frac{\log^{\left[p\right]}M_{g}^{-1}M_{f}\left(r\right)}{\log^{\left[q\right]}r}. \end{array}$$

In fact Definition 4 improves Definition 3 ignoring the restriction *p* ≥ *q*.

In this paper we wish to prove some results related to the growth rates of entire functions on the basis of relative (*p*, *q*)th order and relative (*p*, *q*)th lower order with respect to another entire function extending some earlier results for any two positive integers *p* and *q*.

## 2. Lemmas

In this section we present some lemmas which will be needed in the sequel.

**Lemma 1 (see [7]).**

If *f* and *g* are any two entire functions with *g*(0) = 0. then

(13) Mf∘gr≥Mgr2for  all  sufficiently  large  values  of  r⩾r0.

**Lemma 2 (see [7]).**

Let *f* be entire and let *g* be a transcendental entire function of finite lower order. Then, for any *δ* > 0,

(14) Mf∘gr1+δ≥MfMgrfor  all  sufficiently  large  values  of  r⩾r0.

**Lemma 3 (see [8]).**

If *f* and *g* are any two entire functions with *g*(0) = 0. then, for any 0 < *c* < 1,

(15) Mf∘gr≥MfcMgr2for  all  sufficiently  large  values  of  r⩾r0,

**Lemma 4 (see [9]).**

If *f* and *g* are any two entire functions then for all sufficiently large values of *r*⩾*r*_0_

(16) $$\begin{array}{c} M_{f\circ g}\left(r\right)\leq M_{f}\left(M_{g}\left(r\right)\right). \end{array}$$

## 3. Theorems

In this section we present the main results of the paper.

**Theorem 5 .**

Let *f* be an entire function and let *g* be any polynomial such that *f*∘*g* has got finite relative (*p*, *q*)th order with respect to *h* where *h* is a transcendental entire function and *p*, *q* are any two positive integers. Then *ρ*_*h*_^(*p*, *q*)^(*f*) < *∞*.

**Proof**

Given that *f*∘*g* is of finite relative (*p*, *q*)th order with respect to *h*, we have from Definition 4, for a suitable finite number *μ* > 0 and for all sufficiently large values of *r*, that

(17) $$\begin{array}{c} M_{f\circ g}\left(r\right)<M_{h}\left(\exp^{\left[p\right]}\left(\mu \log^{\left[q\right]}r\right)\right). \end{array}$$

Now let *m* be the order of the polynomial *g* so that

(18) $$\begin{array}{c} g\left(z\right)=c_{1}z+c_{2}z^{2}+\cdots +c_{m}z^{m},\;c_{m}\neq 0. \end{array}$$

Then by Cauchy's inequality we get from (18) that

(19) $$\begin{array}{c} \left|c_{m}\right|r^{m}\leq M_{g}\left(r\right),\;\;\left|z\right|=r. \end{array}$$

Now given 0 < *c* < 1, in view of Lemma 3 and from (17) it follows for all sufficiently large values of *r* that

(20) $$\begin{array}{c} M_{f}\left(c\left|c_{m}\right|{\left(\frac{r}{2}\right)}^{m}\right)\leq M_{f\circ g}\left(r\right)\leq M_{h}\left(\exp^{\left[p\right]}\left(\mu \log^{\left[q\right]}r\right)\right). \end{array}$$

We rewrite the above to the equivalent for all sufficiently large values of *r* that

(21) $$\begin{array}{c} M_{f}\left(r\right)\leq M_{h}\left(\exp^{\left[p\right]}\left(\mu \log^{\left[q\right]}\left({\left(c\left|c_{m}\right|\right)}^{-1}2^{m}r^{1/m}\right)\right)\right). \end{array}$$

Therefore from (21) we get for all sufficiently large values of *r* that

(22) Mh−1Mfr≤exp⁡pμlog⁡qccm−12mr1/m,i.e.,  log⁡pMh−1Mfr≤μlog⁡qccm−12mr1/m.

*Case *I. Assume *q* = 1. Then we have from (22) for all sufficiently large values of *r* that

(23) $$\begin{array}{c} \frac{\log^{\left[p\right]}M_{h}^{-1}M_{f}\left(r\right)}{\log r}\leq \frac{\mu }{m}\frac{\log r+\left|O\left(1\right)\right|}{\log r}, \end{array}$$

where *O*(1) stands for the constant expression, *m*log⁡((*c*|*c*_*m*_|)^−1^2^*m*^). Then

(24) lim⁡sup⁡r→∞log⁡pMh−1Mfrlog⁡r≤μmlim⁡sup⁡r→∞log⁡r+O1log⁡r,i.e.,  ρhpf≤μm<∞.

*Case *II. Let us now assume *q* > 1. Then we obtain from (22) for all sufficiently large values of *r* that

(25) $$\begin{array}{c} \frac{\log^{\left[p\right]}M_{h}^{-1}M_{f}\left(r\right)}{\log^{\left[q\right]}r}\leq \mu \;\frac{\log^{\left[q\right]}r+\left|O\left(1\right)\right|}{\log^{\left[q\right]}r}, \end{array}$$

where *O*(1) stands for a bounded quantity. Then

(26) lim⁡sup⁡r→∞log⁡pMh−1Mfrlog⁡qr≤μ  lim⁡sup⁡r→∞log⁡qr+O1log⁡qr,i.e.,  ρhp,qf≤μ<∞.

Thus the theorem follows from (24) and (26).

In the forthcoming proofs we will assume the natural number *q* to be *q* > 1, the reasonings being easily adapted for *q* = 1.

**Theorem 6 .**

Let *f*, *g*, and *h* be any three transcendental entire functions and let *p* and *q* be two positive integers. If, for any *α*, *β* with 0 < *α* < 1, *β* > 0, and *α*(*β* + 1) > 1, it holds that the two limits *A*, *B* ∈ *R* ^+^ of some of either

- lim⁡sup⁡_*r*→*∞*_(log⁡^[*p*]^ *M*_*h*_^−1^(*M*_*g*_(*r*))/(log⁡^[*q*]^ *r*)^*α*^) = *A*, lim⁡inf⁡_*r*→*∞*_(log⁡^[*p*]^ *M*_*h*_^−1^(*M*_*f*_(*r*))/(log⁡^[*p*]^ *M*_*h*_^−1^(*r*))^*β*+1^) = *B*,
- lim⁡inf⁡_*r*→*∞*_(log⁡^[*p*]^ *M*_*h*_^−1^(*M*_*g*_(*r*))/(log⁡^[*q*]^ *r*)^*α*^) = *A*, lim⁡sup⁡_*r*→*∞*_(log⁡^[*p*]^ *M*_*h*_^−1^(*M*_*f*_(*r*))/(log⁡^[*p*]^ *M*_*h*_^−1^(*r*))^*β*+1^) = *B*, or
- lim⁡inf⁡_*r*→*∞*_(log⁡^[*p*]^ *M*_*h*_^−1^(*M*_*g*_(*r*))/(log⁡^[*q*]^ *r*)^*α*^) = *A*, lim⁡inf⁡_*r*→*∞*_(log⁡^[*p*]^ *M*_*h*_^−1^(*M*_*f*_(*r*))/(log⁡^[*p*]^ *M*_*h*_^−1^(*r*))^*β*+1^) = *B*

exist, then *ρ*_*h*_^(*p*, *q*)^(*f*∘*g*) = *∞*.

**Proof**

(i) The existence of *A* and *B* implies that given any *ɛ* > 0, for sufficiently large values of *r*,

(27) $$\begin{array}{c} \log^{\left[p\right]}M_{h}^{-1}\left(M_{g}\left(r\right)\right)\geq \left(A-ɛ\right){\left(\log^{\left[q\right]}r\right)}^{\alpha },\\ \log^{\left[p\right]}M_{h}^{-1}\left(M_{f}\left(r\right)\right)\geq \left(B-ɛ\right){\left(\log^{\left[p\right]}M_{h}^{-1}\left(r\right)\right)}^{\beta +1}. \end{array}$$

Since *M*_*g*_(*r*) is a continuous, increasing, and unbounded function of *r*, we get from above for all sufficiently large values of *r* that

(28) log⁡pMh−1Mf(Mgr)  ≥B−ɛlog⁡pMh−1Mgrβ+1.

Also *M*_*h*_^−1^(*r*) is an increasing function of *r*; it follows from Lemma 2, (27), and (28) that given *δ* > 0, for a sequence of values of *r* tending to infinity, the following holds:

(29) log⁡pMh−1Mf∘gr1+δ  ≥log⁡pMh−1MfMgr  ≥B−ɛlog⁡pMh−1Mgrβ+1  ≥B−ɛA−ɛlog⁡qrαβ+1,i.e.,    log⁡pMh−1Mf∘gr1+δlog⁡qr1+δ  ≥B−ɛA−ɛβ+1log⁡qrαβ+1log⁡qr1+δ.

Hence

(30) lim⁡sup⁡r→∞log⁡pMh−1Mf∘g(r1+δ)log⁡qr1+δ  ≥lim⁡inf⁡r→∞B−ɛA−ɛβ+1log⁡qrαβ+1log⁡qr+O1

for all sufficiently large values of *r*. Since *ɛ* > 0 is arbitrary and *α*(*β* + 1) > 1 it follows that

(31) $$\begin{array}{c} \rho_{h}^{\left(p,q\right)}\left(f\circ g\right)=\infty . \end{array}$$

Under (ii) or (iii) a similar argument applies.

**Theorem 7 .**

Let *f*, *g*, and *h* be any three transcendental entire functions and let *p* and *q* be two positive integers. If, for any *α*, *β* with *α* > 1, 0 < *β* < 1, and *αβ* > 1, it holds that the two limits *A*, *B* ∈ *R* ^+^ of either

- lim⁡sup⁡_*r*→*∞*_(log⁡^[*p*]^ *M*_*h*_^−1^(*M*_*g*_(*r*))/(log⁡^[*q* + 1]^ *r*)^*α*^) = *A*, lim⁡inf⁡_*r*→*∞*_(log⁡[log⁡^[*p*]^ *M*_*h*_^−1^(*M*_*f*_(*r*))/log⁡^[*p*]^ *M*_*h*_^−1^(*r*)]/[log⁡^[*p*]^ *M*_*h*_^−1^(*r*)]^*β*^) = *B*,
- lim⁡inf⁡_*r*→*∞*_(log⁡^[*p*]^ *M*_*h*_^−1^(*M*_*g*_(*r*))/(log⁡^[*q* + 1]^ *r*)^*α*^) = *A*, lim⁡sup⁡_*r*→*∞*_(log⁡[log⁡^[*p*]^ *M*_*h*_^−1^(*M*_*f*_(*r*))/log⁡^[*p*]^ *M*_*h*_^−1^(*r*)]/[log⁡^[*p*]^ *M*_*h*_^−1^(*r*)]^*β*^) = *B*, or
- lim⁡inf⁡_*r*→*∞*_(log⁡^[*p*]^ *M*_*h*_^−1^(*M*_*g*_(*r*))/(log⁡^[*q* + 1]^ *r*)^*α*^) = *A*, lim⁡inf⁡_*r*→*∞*_(log⁡[log⁡^[*p*]^ *M*_*h*_^−1^(*M*_*f*_(*r*))/log⁡^[*p*]^ *M*_*h*_^−1^(*r*)]/[log⁡^[*p*]^ *M*_*h*_^−1^(*r*)]^*β*^) = *B*

exist, then *ρ*_*h*_^(*p*, *q*)^(*f*∘*g*) = *∞*.

**Proof**

(i) Given any *ɛ* > 0, for a sequence of values of *r* tending to infinity, we get that

(32) $$\begin{array}{c} \log^{\left[p\right]}M_{h}^{-1}\left(M_{g}\left(r\right)\right)\geq \left(A-ɛ\right){\left(\log^{\left[q+1\right]}r\right)}^{\alpha } \end{array}$$

and for all sufficiently large values of *r* that

(33) log⁡log⁡pMh−1Mf(r)log⁡pMh−1r≥B−ɛlog⁡pMh−1rβ,i.e.,  log⁡pMh−1Mf(r)log⁡pMh−1r≥exp⁡B−ɛlog⁡pMh−1rβ.

Since *M*_*g*_(*r*) is a continuous, increasing, and unbounded function of *r*, we get from above for all sufficiently large values of *r* that

(34) log⁡pMh−1Mf(Mgr)log⁡pMh−1Mgr  ≥exp⁡B−ɛlog⁡pMh−1Mgrβ.

Also *M*_*h*_^−1^(*r*) is an increasing function of *r*; thus from Lemma 2, (32), and (34) it follows that, given that *δ* > 0, for a sequence of values of *r* tending to infinity,

(35) log⁡pMh−1Mf∘g(r1+δ)log⁡qr1+δ ≥log⁡pMh−1MfMgrlog⁡qr1+δ ≥log⁡pMh−1MfMgrlog⁡pMh−1Mgr·log⁡pMh−1Mgrlog⁡qr+O1.

Therefore

(36) log⁡pMh−1Mf∘g(r1+δ)log⁡qr1+δ ≥ exp⁡B−ɛlog⁡pMh−1Mgrβ  ·A−ɛlog⁡q+1rαlog⁡qr+O1 ≥exp⁡B−ɛA−ɛβlog⁡q+1rαβ  ·A−ɛlog⁡q+1rαlog⁡qr+O1 =exp⁡B−ɛA−ɛβlog⁡q+1rαβ−1log⁡q+1r  ·A−ɛlog⁡q+1rαlog⁡qr+O1 ≥log⁡qrB−ɛA−ɛβlog⁡q+1rαβ−1  ·A−ɛlog⁡q+1rαlog⁡qr+O1.

Hence

(37) lim⁡sup⁡r→∞log⁡pMh−1Mf∘g(r1+δ)log⁡qr1+δ  ≥ lim⁡inf⁡r→∞log⁡qrB−ɛA−ɛβlog⁡q+1rαβ−1   ·A−ɛlog⁡q+1rαlog⁡qr+O1.

Since *ɛ* > 0 is arbitrary and *α* > 1, *αβ* > 1, it follows that

(38) $$\begin{array}{c} \rho_{h}^{\left(p,q\right)}\left(f\circ g\right)=\infty . \end{array}$$

Under (ii) or (iii) a similar argument may be used.

**Theorem 8 .**

Let *f*, *g*, and *h* be any three transcendental entire functions such that 0 < *λ*_*h*_^(*p*, *q*)^  (*g*) ≤ *ρ*_*h*_^(*p*, *q*)^(*g*) < *∞* where *p* and *q* are any two positive integers. If the limit *A* ∈ *R* exists in either

- lim⁡sup⁡_*r*→*∞*_(log⁡^[*p*]^ *M*_*h*_^−1^(*M*_*f*_(*r*))/log⁡^[*p*]^ *M*_*h*_^−1^(*r*)) = *A* or
- lim⁡inf⁡_*r*→*∞*_(log⁡^[*p*]^ *M*_*h*_^−1^(*M*_*f*_(*r*))/log⁡^[*p*]^ *M*_*h*_^−1^(*r*)) = *A*,

then

(39) $$\begin{array}{c} \lambda_{h}^{\left(p,q\right)}\left(f\circ g\right)\leq A\lambda_{h}^{\left(p,q\right)}\left(g\right)\leq \rho_{h}^{\left(p,q\right)}\left(f\circ g\right)\leq A\rho_{h}^{\left(p,q\right)}\left(g\right). \end{array}$$

**Proof**

(i) Since *M*_*h*_^−1^(*r*) is an increasing function of *r*, it follows from Lemmas 2 and 4, given *δ* > 0, for all sufficiently large values of *r*, that

(40) $$\begin{array}{c} M_{h}^{-1}M_{f\circ g}\left(r^{1+\delta }\right)\geq M_{h}^{-1}\left\{M_{f}\left(M_{g}\left(r\right)\right)\right\}, \end{array}$$

(41) $$\begin{array}{c} M_{h}^{-1}M_{f\circ g}\left(r\right)\leq M_{h}^{-1}\left\{M_{f}\left(M_{g}\left(r\right)\right)\right\}, \end{array}$$

respectively.

Therefore from (40) we get for all sufficiently large values of *r* that

(42) log⁡pMh−1Mf∘g(r1+δ)log⁡qr1+δ≥log⁡pMh−1MfMgrlog⁡qr1+δ≥log⁡pMh−1MfMgrlog⁡pMh−1Mgr ·log⁡pMh−1Mgrlog⁡qr+O1.

From here it follows that

(43) lim⁡sup⁡r→∞log⁡pMh−1Mf∘g(r)log⁡qr ≥ lim⁡sup⁡r→∞log⁡pMh−1MfMgrlog⁡pMh−1Mgr  ×lim⁡inf⁡r→∞log⁡pMh−1Mgrlog⁡qr+O1i.e.,  ρhp,qf∘g≥Aλhp,qg.

Similarly from (41) it follows for all sufficiently large values of *r* that

(44) $$\begin{array}{c} \log^{\left[p\right]}M_{h}^{-1}M_{f\circ g}\left(r\right)\leq \log^{\left[p\right]}M_{h}^{-1}\left(M_{f}\left(M_{g}\left(r\right)\right)\right). \end{array}$$

Therefore

(45) log⁡pMh−1Mf∘grlog⁡qr ≤log⁡pMh−1MfMgrlog⁡pMh−1Mgr·log⁡pMh−1Mgrlog⁡qr.

Hence

(46) lim⁡inf⁡r→∞⁡log⁡pMh−1Mf∘grlog⁡qr ≤ lim⁡sup⁡r→∞log⁡pMh−1MfMgrlog⁡pMh−1Mgr  ×lim⁡inf⁡r→∞log⁡pMh−1Mgrlog⁡qr,i.e.,  λhp,qf∘g≤Aλhp,qg.

Also from (45) we obtain for all sufficiently large values of *r* that

(47) lim⁡sup⁡r→∞log⁡pMh−1Mf∘g(r)log⁡qr  ≤lim⁡sup⁡r→∞log⁡pMh−1MfMgrlog⁡pMh−1Mgr   ×lim⁡sup⁡r→∞log⁡pMh−1Mgrlog⁡qr,i.e.,  ρhp,qf∘g≤A·ρhp,qg.

Then the thesis follows from (43), (46), and (47).

(ii) follows with a similar argument.

**Theorem 9 .**

Let *f*, *g*, and *h* be any three transcendental entire functions with *g*(0) = 0. If *p*, *q*, and *m* are any three positive integers with *m* > *q*, then *ρ*_*h*_^(p, *q*)^(*f*∘*g*) = *∞* under any of the following conditions:

- *ρ*_*h*_^(*p*, *q*)^(*g*) = *∞*;
- min⁡(*ρ*_*h*_^(*p*, *q*)^(*f*), *λ*_*g*_(*m*, *q*)) > 0;
- min⁡(*ρ*_*g*_(*m*, *q*), *λ*_*h*_^(*p*, *q*)^(*f*)) > 0.

**Proof**

(i) If *ρ*_*h*_^(*p*, *q*)^(*g*) = *∞*, since *M*_*h*_^−1^(*r*) is an increasing function of *r*, it follows from Lemma 1, for all sufficiently large values of *r*, that

(48) $$\begin{array}{c} \log^{\left[p\right]}M_{h}^{-1}\left(M_{f\circ g}\left(r\right)\right)\geq \log^{\left[p\right]}M_{h}^{-1}\left(M_{g}\left(\frac{r}{2}\right)\right). \end{array}$$

Therefore

(49) log⁡pMh−1Mf∘g(r)log⁡qr≥log⁡pMh−1Mgr/2log⁡qr≥log⁡pMh−1Mgr/2log⁡qr/2+O1.

Then

(50) lim⁡sup⁡r→∞log⁡pMh−1Mf∘g(r)log⁡q  ≥lim⁡sup⁡r→∞log⁡pMh−1Mgr/2log⁡qr/2+O1,i.e.,  ρhp,qf∘g≥ρhp,qg=∞.

(ii) Suppose *ρ*_*h*_^(*p*, *q*)^(*f*) > 0 and *λ*_*g*_(*m*, *q*) > 0.

As *M*_*h*_^−1^(*r*) is an increasing function of *r*, we get from Lemma 2 that given *δ* > 0 and any *ɛ* > 0, for all sufficiently large values of *r*,

(51) log⁡pMh−1Mf∘gr1+δ  ≥log⁡pMh−1MfMgr  ≥ρhp,qf−ɛlog⁡qMgr  ≥ρhp,qf−ɛexp⁡m−q−1log⁡q−1rλgm,q−ɛ.

Thus

(52) log⁡pMh−1Mf∘g(r1+δ)log⁡qr1+δ ≥ρhp,qf−ɛexp⁡m−q−1log⁡q−1rλgm,q−ɛlog⁡qr1+δ.

Hence

(53) lim⁡sup⁡r→∞log⁡pMh−1Mf∘g(r1+δ)log⁡qr1+δ≥ lim⁡inf⁡r→∞ρhp,qf−ɛexp⁡m−q−1log⁡q−1rλgm,q−ɛlog⁡qr+O(1),i.e.,  ρhp,qf∘g=∞.

Under (iii) a similar argument to (i) applies.

In the line of Theorem 9 one can easily prove the following result.

**Theorem 10 .**

Let *f*, *g*, and *h* be any three transcendental entire functions with *g*(0) = 0. If *p*, *q*, and *m* are any three positive integers with *m* > *q*, then *λ*_*h*_^(*p*, *q*)^(*f*∘*g*) = *∞* if any of the following facts happens:

- *λ*_*h*_^(*p*, *q*)^(*g*) = *∞*;
- min⁡(*λ*_*h*_^(*p*, *q*)^(*f*), *λ*_*g*_(*m*, *q*)) > 0.

**Theorem 11 .**

Let *f*, *g*, and *h* be any three transcendental entire functions such that *g*(0) = 0. If *p*, *q*, and *m* are any three positive integers with *m* > *q* and any of the following two facts happens

- min⁡(*ρ*_*h*_^(*p*, *q*)^(*f*), *λ*_*g*_(*m*, *q*)) > 0 or
- min⁡(*λ*_*h*_^(*p*, *q*)^(*f*), *λ*_*g*_(*m*, *q*)) > 0,

then

(54) $$\begin{array}{c} \lim\underset{r\to \infty }{\sup}\frac{\log^{\left[p\right]}M_{h}^{-1}\left(M_{f\circ g}\left(r\right)\right)}{\log^{\left[p\right]}M_{h}^{-1}\left(M_{f}\left(r\right)\right)}=\infty . \end{array}$$

**Proof**

(i) Since

(55) lim⁡sup⁡r→∞log⁡pMh−1Mf∘g(r)log⁡pMh−1Mf(r)  ≥ lim⁡sup⁡r→∞log⁡pMh−1Mf∘g(r)log⁡qr   ×lim⁡inf⁡r→∞log⁡qrlog⁡pMh−1Mf(r)  =ρhp,qf∘g  1ρhp,qf

the result follows from Theorem 9.

(ii) The proof can be carried out in the line of (i) and Theorem 10.

## 4. Conclusion

After modifying the notion of relative order of higher dimensions in case of entire functions in [6], where a number of examples of relative order between functions were provided, in this paper we have obtained some growth properties of composite entire functions on the basis of relative (*p*, *q*)th order and relative (*p*, *q*)th lower order. In this process, Theorem 5 and the first part of Theorem 6 and Theorems 7 and 8 can be regarded as extensions of some results of [10].
